# Sericultural By-Products: The Potential for Alternative Therapy in Cancer Drug Design

**DOI:** 10.3390/molecules28020850

**Published:** 2023-01-14

**Authors:** Gabriela-Maria Baci, Ecaterina-Daniela Baciu, Alexandra-Antonia Cucu, Adriana-Sebastiana Muscă, Alexandru Ioan Giurgiu, Adela Ramona Moise, Marius Zăhan, Daniel Severus Dezmirean

**Affiliations:** Faculty of Animal Science and Biotechnology, University of Animal Sciences and Veterinary Medicine Cluj-Napoca, 400372 Cluj-Napoca, Romania

**Keywords:** silkworms, silk fibroin, biomaterials, mulberry, cancer therapy, 3D models

## Abstract

Major progress has been made in cancer research; however, cancer remains one of the most important health-related burdens. Sericulture importance is no longer limited to the textile industry, but its by-products, such as silk fibroin or mulberry, exhibit great impact in the cancer research area. Fibroin, the pivotal compound that is found in silk, owns superior biocompatibility and biodegradability, representing one of the most important biomaterials. Numerous studies have reported its successful use as a drug delivery system, and it is currently used to develop three-dimensional tumor models that lead to a better understanding of cancer biology and play a great role in the development of novel antitumoral strategies. Moreover, sericin’s cytotoxic effect on various tumoral cell lines has been reported, but also, it has been used as a nanocarrier for target therapeutic agents. On the other hand, mulberry compounds include various bioactive elements that are well known for their antitumoral activities, such as polyphenols or anthocyanins. In this review, the latest progress of using sericultural by-products in cancer therapy is discussed by highlighting their notable impact in developing novel effective drug strategies.

## 1. Introduction

Worldwide, cancer is one of the major health and economic burdens. Even if significant progress has been made in cancer research and there have been described new treatments, nevertheless, cancer continues to be one of the leading causes of death [1,2,3,4]. With the purpose to overcome specific limitations of the current cancer treatments, there have been numerous biomaterials developed, such as polymers, ceramics, or biologically derived materials. Among these, the most used in the process of fighting cancer, due to their high biocompatibility, are biologically derived materials [5]. Using biomaterials to combat cancer, numerous studies have shown great progress in reducing chemotherapeutics’ toxicity, expanding its half-life, and also in improving anticancer drugs’ solubility [6]. Moreover, in numerous experiments, biomaterials are used to develop tumor microenvironment models to elucidate certain cancer mechanisms that would lead to the development of novel therapies [7].

Another promising strategy to prevent and to treat cancer is the use of specific plants and plant-derived products as anticancer agents. This strategy also includes the exploitation of the chemoprotective activity of a wide range of plants to increase the cancer patient’s life quality [8]. The most important plant-derived compound that is currently used as a first-line treatment in different types of cancer is paclitaxel, which is extracted from *Taxus chinensis* [9,10,11]. Furthermore, this strategy has brought into the spotlight a broad range of plants that anticancer activity has been certified, for instance: *Curcuma longa*, *Artemisia annua*, *Peganum harmala* [10], and *Morus alba* [12,13], just to mention a few.

Nowadays, one of the most important agro-based industries is sericulture. The main activity of sericulture (*Bombyx mori*) is the rearing of silkworms in order to obtain raw silk. For example, the rearing of silkworms (*Antheraea pernyi*) in China is to obtain pupae and moths for food and raw silk for textiles [14]. However, sericulture (*B. mori*) also involves the cultivation of food plants to provide silkworms’ nourishment source. Furthermore, it includes silk yarn extraction and a wide range of post-cocoon activities [15,16]. There are several species of silkworms that have distinct nutritional requirements and produce different types of silk. By far, among the silkworm species, *B. mori* exhibits the greatest importance, not only in terms of the silk industry but it displays an extraordinary impact in the life science fields [15,17,18,19]. Along with *Apis mellifera* and *Drosophila melanogaster* [20], the silkworm, *B. mori*, represents one of the most important insects that exhibits considerable interest for the scientific community, being extensively studied in worldwide research laboratories [21]. Its potential in life science areas began to be massively exploited in 2004 when its draft genome sequence was published [22]. One of the most important roles of *B. mori* in the scientific fields is that of a model organism and bioreactor. It represents a valuable model organism for various human diseases due to the fact that its genetic information is well documented [23]. In addition, the silkworm’s genome contains a meaningful percent of human ortholog genes, and there are various mutant strains described [21].

However, the scientific community’s interest in silkworms is also highlighted by the key role of silk fibroin (SF) as a pivotal biomaterial [24]. Fibroin is the main compound of the silk produced by *B. mori*. It possesses certain properties, such as superior biocompatibility, biodegradability, and remarkable mechanical features, that have led to the development of an extensive variety of SF-based biomaterials [25,26,27,28]. There have been various types of SF biomaterials described [29], such as hydrogels [30,31,32,33], scaffolds [34,35,36,37], and sponges [38,39,40,41]. On the other hand, numerous studies have reported the antitumoral effect of sericin and its impact as a nanocarrier biomaterial for anticancer therapeutic agents [42].

*B. mori*’s unique nourishment source is represented by mulberry leaves [15,18,43,44]. Nevertheless, due to its complex composition since ancient times, it has been recognized as a significant medicinal plant, and nowadays, mulberry represents one of the pivotal functional foods. Among mulberry ingredients, the most notable elements, which are responsible for a broad spectrum of bioactivities, are the phenolic compounds [45]. There are numerous epidemiologic studies that have certified mulberry plant’s medicinal value—more specifically, its anticancer, chemoprotective, antioxidative, cardioprotective, anti-obesity, and anti-inflammatory activities [45,46,47,48,49,50,51,52,53,54].

Nutrition directly influences larval growth, such as the economic traits, including filament length and fibroin content. It is well documented that the leaf quality and its nutritional value impact silk production [55]. Nowadays, by keeping in mind the leading role of SF as a biomaterial in cancer research and beyond, it is of keen interest to enhance the fibroin content. Considering this, the scientific community paid considerable attention to supplementing silkworms’ traditional food. For instance, Thangapandiyan and Dharanipriya (2019) [56] investigated the influence of the synergistic effect of spirulina and silver nanoparticles on a wide range of *B. mori*’s economic traits, including the SF and sericin contents. Their results showed that the chosen agents positively impacted the cocoon’s characters. In other studies, superior quality silk fiber was obtained by supplementing mulberry leaves with pollen [57], honey [58], lactic acid [59], palm sugar [60], micronutrients [61], or the Amway protein [62].

Keeping in mind the significant need of developing new strategies to combat cancer, it is of great importance to point at the progress and perspectives of the functional aspects of sericulture in cancer therapy. Herein, we discuss the latest progress that has been made in developing fibroin-based biomaterials to overcome certain limits of chemotherapeutic drugs but also in using this type of biomaterial to mimic the tumor microenvironment. Furthermore, we summarize the perspective of exploiting mulberry’s anticancer activity to develop novel cancer drug designs.

## 2. Sericultural By-Products

**Figure 1:**
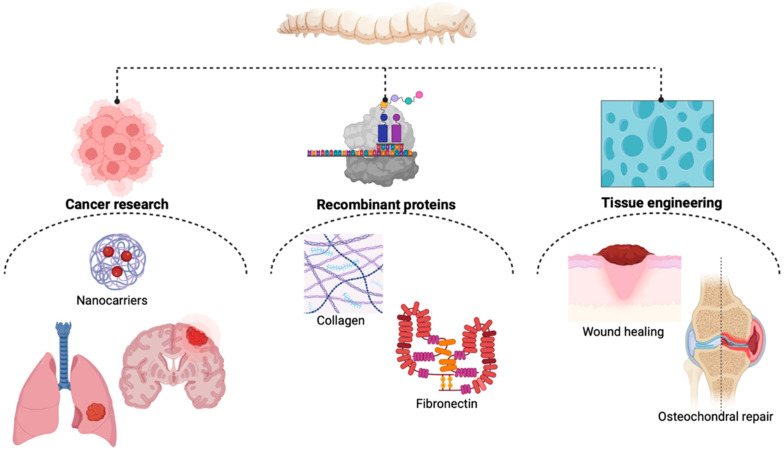
*B. mori*’s applicability in biomedicine (Created with BioRender.com, accessed on 23 November 2022)).

In recent years, the concept of sericulture has not been limited anymore to the silk industry. The great medicinal potential of silkworms (Figure 1), SF, and mulberry plants have brought sericulture to researchers’ attention [63].

### 2.1. Silk Fibroin as a Functional Biomaterial

**Figure 2:**
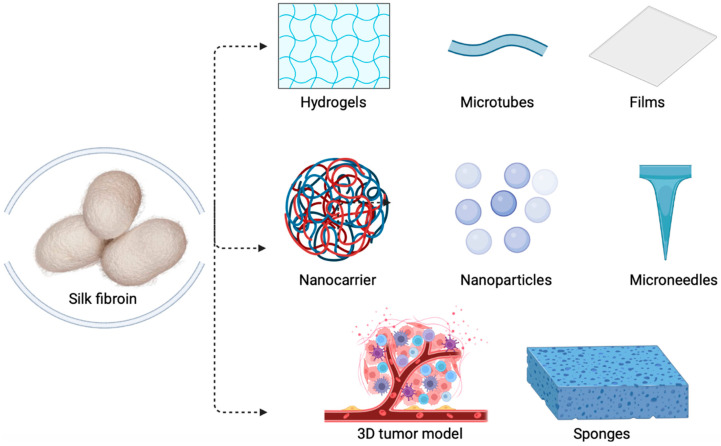
Silk fibroin-based biomaterials (Created with BioRender.com, accessed on 23 November 2022)).

One of the most appreciated naturally derived polymers, among cellulose and chitosan, is silk. Numerous insects produce this biopolymer; however, the most explored is the silk produced by the silkworm *B. mori* [64]. Owning extraordinary properties such as superior mechanical strength, flexibility, and breathability, it is one of the most important luxury fibers [65]. Moreover, the broad range of qualities have brought silk to researchers’ attention, and other great features of silk have been documented—more specifically, its great biocompatibility, versatility, and biodegradability. Based on these features, silk has been used as a surgical suture since ancient times [66,67]. With reference to the structural composition of *B. mori*’s silk, it includes two major players, namely fibroin and sericin. In the silk thread, there are two fibroin fibers, while sericin represents a glue-like protein for them [64]. Fibroin is responsible for silk’s superior properties; in opposition, in most cases, sericin is removed by certain thermos–chemical treatments [68]. At this time, fibroin is being explored by a wide range of researchers that develop various SF-based biomaterials, such as hydrogels, nanoparticles, scaffolds, microtubes, biofilms, and sponges (Figure 2), which exhibit great importance for the life science fields [69,70].

Gastrointestinal disorders are one of the most common chronic conditions; accordingly, there is an extensive need to prevent these diseases [71]. In this direction, one of the most approached strategies is represented by probiotics. In the past years, the use of probiotics met rapid enlargement, even though their history began centuries ago. Probiotics consist of living microorganisms that exhibit various benefits for human health [72,73], especially for certain gastrointestinal disorders. In addition, the health-promoting properties are not limited to gastrointestinal diseases, and it has been certified that probiotics are beneficial to prevent and to treat oral cavity and skin disorders [74]. However, specific tissues and organs exhibit certain limitations when it comes to probiotics’ viability and activity; specifically, there are disturbing factors such as oxidative stress or pH. The most used strategy to overcome the viability and activity limitations is the probiotics’ encapsulation by using biomaterials [75,76].

Recently, Kwon et al. (2021) [27] used SF as a coating biomaterial for various probiotics. By using the Coeuret’s method, the authors isolated the target bacterial from newborn infant feces, fermented fruits, and raw milk. Regarding the SF-coating preparation, the silk threads were firstly degummed to remove sericin, and subsequently, a treatment with two agents (Protmax and T100) was applied to enhance SF’s solubility but also its absorption rate. Primarily, the authors investigated if the addition of water-soluble calcium to SF-coated bacterial strains impacted its survival ratio. Additionally, to evaluate the effects of using SF as a coating biomaterial, the gastrointestinal conditions were stimulated to analyze the acid and bile tolerance. The SF-coated probiotics were exposed to simulated gastric fluid, which included HCl and NaOH for pH adjustments and 0.5% oxgall. The indirect method of evaluating the intestinal adhesion of probiotics implies the determination of cell surface hydrophobicity. Moreover, the researchers directly investigated probiotics’ intestinal adherence by using a human colorectal adenocarcinoma cell line. By evaluating the impact of several different concentrations of water-soluble calcium on a bacterial strain’s survival rate, their results revealed that the highest survival ratio was observed when adding 0.1% of this component. Successfully, by using SF as coating biomaterial for probiotics, the survival rate was positively impacted under a simulated gastrointestinal environment. When simulating the gastric fluid, the target parameter was enhanced by 13.3–31.3%, and regarding the simulated intestinal fluid, the bacterial survival rate was increased by 4.8–23.5%. Their data showed that SF coating led to a significant increase of the cell surface hydrophobicity; notably, the highest increase observed was up to 71.7%. Furthermore, when evaluating the cell adhesion capacity, the authors reported that it has been enhanced up to 36%. Their data highlight the positive effects of using SF as a coating biomaterial on probiotic bacterial stability. In the specialized literature, there are a broad range of recent studies that have reported the successful use of SF as a biomaterial for various purposes (Table 1).

### 2.2. Therapuetic Potential of Mulberry

In the healthcare system, a great number of plants are engaged as curative agents and functional foods, mainly due to the great number of bioactive compounds that are found in plants [98]. Furthermore, these organisms have received considerable attention, because various parts of the plants can be exploited for medical purposes, the most notable being the leaves, fruits, seeds, and roots. In addition, another great advantage when it comes to therapeutic herbs is their low cost [99,100].

**Figure 3:**
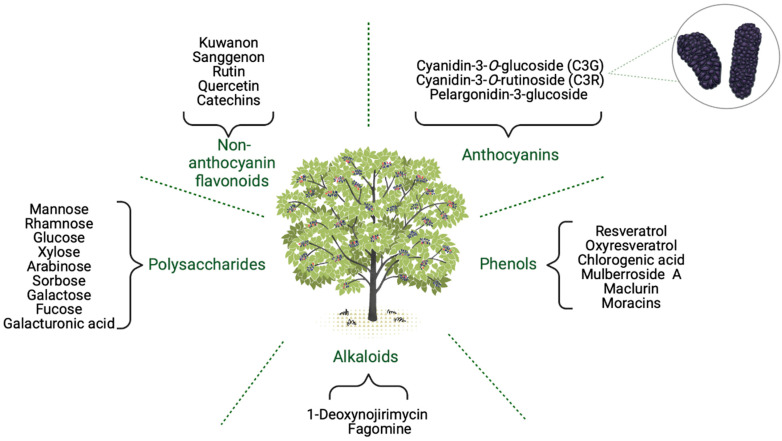
Main bioactive compounds in the mulberry (Created with BioRender.com, accessed on 23 November 2022).

Among all powerful therapeutic plants, such as Allium sativum [101], Citrus limon [102], and Vaccinium corymbosum [103], one of the richest plants in bioactive components is the mulberry (Morus spp.). A generous spectrum of recent studies highlighted the beneficial effects of various parts of the mulberry on human health [104,105]. Each botanical part of the mulberry tree exhibits certain therapeutic effects—more specifically, the mulberry fruits, leaves, roots, and twigs [106]. As for mulberry’s composition, it includes a great content of carbohydrates (7.8–9%) and proteins (5–1.4%) [104] but also lipids, fibers, vitamins, and minerals [107]. In the matter of the functional compounds, in Figure 3 are listed the main bioactive elements found in mulberries that are associated with powerful therapeutic effects. Notably, the chemical composition of the mulberry depends on the mulberry variety, climate, and soil condition [104].

Oxidative stress is one of the major threats in cellular damage being responsible for aging, cardiovascular diseases, cancer, and so on [108]. In this direction, a certain group of plants’ secondary metabolites, namely the phenolic compounds, have gained a lot of attention by playing a key role as antioxidative agents. These secondary metabolites are extensively present in various plant foods, such as apples, cranberries, and pears [109,110]. Another plant that exhibits a great number of phenolic compounds is the mulberry. Numerous studies have investigated the phenolic profiles of numerous varieties of the mulberry and confirmed its antioxidative activities [111,112,113,114].

In a recent study, Hu et al. (2021) [115] investigated wherever the mulberry growth period impacts the number of flavonoids in leaves. The authors also explored the influence of several distinct drying methods on the flavonoid levels. Moreover, the flavonoid content was correlated with the antioxidative activity of mulberry leaves. Their data revealed that the flavonoids’ levels and the antioxidative effect are impacted by both variables. When it comes to the mulberry growth time, the highest flavonoid accumulation was registered in two situations, namely in the leaves that grew for a long period of time and in the leaves that were not exposed long term to environmental stress. As for the evaluated drying methods, namely the sun, air, oven, and freeze drying, their results showed that, by using the first two methods, the highest flavonoid content was registered. On the other hand, by using air-drying and freeze-drying methods, the antioxidative effects of the flavonoids were better retained.

Jaruchotikamol and Pannangpetch (2013) [116] analyzed the cytoprotective action of M. alba leaf extract. Firstly, in vitro, the mulberry leaves extract exhibited a scavenging action on a certain free radical, namely 1, 1-diphenyl-2-picrylhydrazyl (DPPH). The authors revealed that the antioxidative effect of mulberry leaves depends on the concentration. However, the authors used vitamin C as a positive control and showed that, even if mulberry extract possesses certain antioxidative effects, vitamin C displays more powerful antioxidative activity. Additionally, by using 2, 2′-azobis (2-amidinopropane) dihydrochloride (AAPH), they induced hemolysis in order to investigate the protective action of the mulberry extract on erythrocytes. Positively, the target extract registered protective action against hemolysis induced by reactive oxygen species. After confirming the therapeutic effect of the mulberry extract in vitro, the authors further investigated it in vivo, in rats, by inducing a gastric mucosal lesion through ischemia–reperfusion (I/R). For three days, the rats received oral doses of mulberry extract of different quantities: 0.25, 0.50, 1, and 2 g/kg/day, respectively. Interestingly, by administering 0.25 and 0.50 g/kg/day doses, the target product exposes great protective action against gastric mucosal injury. To the contrary, when higher doses were administered, the protective action of the investigated extract was absent.

In Table 2 are listed various recent studies that certified mulberry’s health-promoting potential.

## 3. Fibroin’s Impact in Anticancer Therapy

### 3.1. Fibroin-Based Target Delivery Systems

Nowadays, tremendous progress has been made in overcoming the limitations of conventional cancer therapies. The most used therapeutic modalities in cancer are chemotherapy and radiotherapy. It is well documented that these treatments include a broad range of negative side effects, such as systemic toxicity and psychological problems, and also, the tumor cells develop high drug resistance [125]. One great strategy to overcome both the drug resistance and negative side effects is the use of certain drug delivery systems [126].

In order to develop a target delivery system, Mottaghitalab et al. (2017) [127] used loaded SF nanoparticles to target lung tumors. The prepared SF nanoparticles were loaded with gemcitabine and SP5-52 peptide. The last compound was conjugated due to its ability to conduct the anticancer drug carriers, and by adding it, a target delivery system was obtained. The prepared SF nanoparticles were acquired by using the prepared SF solution to dissolve 10 mL of DMSO. After it was centrifuged, the precipitate nanoparticle aggregates were evaluated to determine its size, morphology, and surface charge by using both dynamic light scattering and scanning electron microscopy. The peptide Sp5-52 was covalently attached to the loaded SF nanoparticles by using two elements, namely 1-ethyl-3-(3-dimethyl aminopropyl) carbodiimide and N-hydroxysuccinimide. Following the addition of 25 mg of the obtained nanoparticles and 2.5 mg of gemcitabine into 10 mL DMSO, the solution was sonicated and dialyzed against water. UV spectroscopy was used to determine the quantity of the loaded drug. The same technique was used to examine the gemcitabine release profile. Additionally, the toxicity of the loaded nanoparticles was tested both in vitro and in vivo by using lung carcinoma cells and a normal lung epithelial cell line and mice, respectively. Regarding the in vivo experiments, the authors induced lung tumors in mice. Their data revealed that, by adding SP5-52 peptide to the SF gemcitabine-loaded nanoparticles, superior cytotoxicity and cellular uptake was observed, compared with the single drug-loaded nanoparticles and control group. Furthermore, by using this delivery strategy, in the lung tissue, a greater accumulation of gemcitabine was recorded. The in vivo evaluation showed that the targeted delivery system led to higher mice survival rates and a lower mortality percent. No metastasis was observed in mice after the target treatment was applied. This study underlines the efficacy of using SF as a drug delivery system.

Zare-Zardini et al. (2021) [128] developed two SF-based biomaterials as drug delivery approaches, specifically micro- and nanoparticles. The fibroin carriers were loaded with doxorubicin, and its biological activity was assessed in this formulation. The cytotoxicity of the coated therapeutic agent was confirmed by using three different cell lines, specifically breast cancer, normal, and bone cancer cell lines. Two techniques, staining with PI/annexin V and flow cytometry, respectively, were used in order to determine the induced apoptosis, and the results revealed that, by using both developed doxorubicin delivery systems, notable levels of apoptosis were observed. Additionally, the authors evaluated the *p53* gene expression by using real-time PCR, and their data showed that the doxorubicin-loaded fibroin nanoparticles led to a remarkable increase in the *p53* expression level in both tumoral cell lines; on the other hand, a notable decrease was observed in the healthy cell line. Employing the doxorubicin microparticles in normal and breast cancer cell lines, an increased level of expression of *p53* was observed; however, in the osteogenic sarcoma cell line, the expression level decreased. These strategies exhibit great potential as new antitumoral treatments.

The impact of using SF as a drug delivery platform was reported by Wu et al. (2018) [129]. The authors targeted the cancer stem cells that exhibit chemotherapy resistance. It has been certified that salinomycin, a veterinary antibiotic, is effective against cancer stem cell proliferation. Keeping in mind that salinomycin poisoning is lethal, the authors used SF nanoparticles to achieve drug-controlled delivery in order to administer salinomycin in a safer and more efficient manner. Several studies demonstrated that this specific antibiotic sensitized paclitaxel, a well-known chemotherapeutic agent [130,131]. Furthermore, they also prepared paclitaxel-loaded nanoparticles to develop a codelivery system for anticancer treatment. After preparing the SF salinomycin and paclitaxel-loaded nanoparticles, the authors fabricated a SF-based hydrogen and incorporated the nanoparticles into it. Prior to SF gelation, the loaded nanoparticles were dispersed into it. The hydrogel’s biodegradability and its injectable properties were maintained. Additionally, when analyzing the drug distribution, the authors observed that, by incorporating drug-loaded nanoparticles into the hydrogel, the distribution was more homogeneous compared to the hydrogel, which did not have incorporated nanoparticles but uncoated drugs. In terms of tumor growth inhibition, by comparing the codelivery strategy with the single drug treatment in the murine hepatic carcinoma tumor model, the first mentioned therapy resulted in superior tumor growth inhibition. In addition, the developed fibroin-based strategy increased the salinomycin tolerance.

In a novel study, Suyamud et al. (2021) [132] used SF as a coating biomaterial for doxorubicin-loaded liposomes. SF was chosen for liposomal coating to prolong doxorubicin’s circulation time but also to improve its long-term efficacy. The main objectives of this study were to evaluate the encapsulation efficiency, the anticancer effect, the cytotoxicity in healthy cells, and, also, the doxorubicin release profile. The authors examined the anticancer activity by using two different breast cancer cell lines. Their data showed that, by using fibroin as the coating biomaterial, the anticancer effect was diminished; however, the author achieved doxorubicin long-term doxorubicin release. Moreover, by using SF, the cytotoxicity of doxorubicin on healthy cells was reduced.

In another study [133], SF was used as a nanocarrier for curcumin, a well-known phenolic pigment that exhibits extraordinary biological activities, including an antitumoral effect. Even if it displays great antitumoral activity, there are several major impediments regarding its bioavailability, including rapid intestinal metabolism and low water solubility. Accordingly, it is of great importance to use nanocarriers to foster the delivery of curcumin. The loaded SF-based nanoparticles were developed by using two techniques, specifically coprecipitation and physical adsorption. The cytotoxic effect of formulated nanoparticles was evaluated on normal, neuroblastoma, and hepatocellular carcinoma cell lines. The in vitro toxicity experiments confirmed the antitumoral effect of curcumin-loaded SF nanoparticles on cancer cell lines but not on the normal cell lines. This study confirmed the chemotherapeutic and chemopreventive potential of curcumin but also the feasibility of using SF as a nanocarrier. In Table 3 are recorded the novel studies that used various fibroin-based carriers for a cancer therapeutic approach.

### 3.2. Fibroin-Based Tumor Models

One of the most important strategies that leads to a better understanding and plays a great role in fighting cancer is the development of three-dimensional (3D) tumor models. Its production overcomes the limitations of using animal models that cannot mimic numerous human aspects, including the immune system, stromal interactions, and metabolism [153]. By using these tools, numerous cancer aspects can be studied, such as cancer cell differentiation, proliferation, angiogenesis, drug resistance, immunosurveillance, testing new therapeutics, and so on [154]. There are numerous biomaterials that are currently being used for developing these bioengineered platforms, including alginate, chitosan, SF, agarose, and fibrinogen [155]. Due to its biocompatibility and biodegradability, fibroin is one of the most used biomaterials for the development of 3D tumor models [156].

Buhome et al. (2022) [157] developed SF gelatin/hyaluronic acid/heparan sulfate scaffolds (SF-GHH) that served as 3D models in order to facilitate and accelerate the investigation of the role played by cancer stem cells in cholangiocarcinoma. In this study, the authors used the developed scaffold to examine the cancer cell morphology and proliferation. The tumor microenvironment (SF-GHH) was fabricated in various blending ratios (2:1, 1:1, and 1:2) by lyophilization, and their data showed that, by using a ratio of 1:2, the scaffold could simulate the extracellular matrix. In terms of its physical features, including the optimal porosity, stable beta-sheet, and water uptake characteristics, the best results were observed by using a scaffold with a 350 ± 102 μm pore size. The SF-GHH 3D model supported cell proliferation, cell aggregation, and attachment, which led to spheroid formation. Moreover, the authors investigated the expression levels in the 3D culture of two stemness-related genes, specifically *Sox2* and *Nanog*, but also of four epithelial–mesenchymal transition-related genes: *MMP-9*, *Snail-1*, *Twist1*, and *Zeb1*. The results showed that, by using a SF-GHH scaffold as a 3D model, the targeted genes were upregulated.

In a recent study, SF-based hydrogels were used as artificial biomimetic 3D matrices. The authors developed rapidly responsive hydrogels, keeping in mind the great impact of proteins’ conformational transition on cell behavior and tumor suppression. By using the horseradish peroxidase and hydrogen peroxide crosslinking method, the researchers obtained rapidly responsive SF-based hydrogels that, under physiological conditions, exhibited conformational changes; specifically, random coil SF were converted in β-sheet SF hydrogels. A very interesting characteristic of the SF-based developed hydrogel was the conformation influence on hydrogels’ opaque morphology; more specifically, a transparent appearance was observed during the random coil conformation and was opaque during the β-sheet form. For the in vitro evaluation, the hydrogels were used for human neuronal glioblastoma cell encapsulation, showing that the transparent hydrogels stimulate cell viability while the opaque SF hydrogels induced cell apoptosis. These findings exhibit major progress on investigating programed cancer cells death [158].

Dondajewska et al. (2017) [159] used SF to develop porous scaffolds to investigate breast tumor biology but also to analyze the antitumoral activity of doxorubicin. In the developed SF scaffolds were seeded two different cell lines, EMT6 breast cancer and NIH3T3 fibroblast, respectively, in order to study cell–cell interactions but also cell–extracellular matrix relations. Two specific methods, namely lyophilization and salt leaching, were used to develop four distinct types of scaffolds. The developed biomaterials exhibited differences in terms of pore size and shape and also scaffold thickness. By using the salt leaching method, the authors observed spherical pores that were uniformly distributed; on the other hand, by using the lyophilization technique, longitudinal pores were detected. The cell attachment was not impacted by the used technique; however, the cancer line attached slower to the silk scaffold compared to fibroblasts. These results confirm the fact that SF supports cell cultures. Furthermore, their data showed that the cell lines that were grown in 3D cultures were more resistant to the antitumoral activity of doxorubicin compared to its effects in 2D cultures. This underlines the potential of using a SF tumor model to analyze the anticancer drug agent’s ability to penetrate the tumor.

In another novel study (2022) [160], SF and chitosan were associated with developing 3D scaffolds for chemotherapeutic drug screening sensitivity. Two cross-linking elements were used to prepare the target scaffolds—specifically, 1-ethyl-3-(3-dimethylaminopropyl)-carbodiimide and sodium tripolyphosphate—and various techniques such as scanning electron microscopy, swelling, or the water absorption ration were used to characterize its properties. A colon cancer cell line (LoVo) was used to investigate scaffold’s impact on cell adhesion and growth. In addition, a breast cancer cell line, MDA-MB-231, was employed for drug sensitivity testing. The authors reported that, by using 3D scaffolds, a superior sensitivity of the drug screening process was observed compared to the results registered when using 2D models.

Another research group used SF and chitosan to develop a valuable platform for mimicking the tumor microenvironment in order to evaluate the activity of certain anticancer drugs. Human tumoral lung cell line (A549) was seeded on a SF/chitosan porous scaffold and displayed good distribution after 24 h, and after 48 h, cancer cells displayed a spherical shape and were also interconnected. Moreover, after 72 h, a high rates of tumor spheres were observed, and their sizes increased. Compared to 2D models, by using 3D SF/chitosan scaffolds, the tumoral cell line had a superior adhesion, distribution, and proliferation rate. Doxorubicin was used to evaluate the drug resistance of target tumoral cells on the porous scaffold. Their results showed that, by using a 3D model, a higher concentration of chemotherapeutic agent was needed for 50% cell death compared to the 2D systems. This underlines the drug incapacity of uniformly diffusing and the necessity of studying the tumor microenvironment in the process of drug design. Their data showed that, by using SF/chitosan scaffolds, various valuable pieces of information can be brought to light regarding the anticancer activity of specific chemotherapeutic agents and tumor drug resistance [161].

## 4. Sericin in Cancer Research

Although SF is the main protein used in cancer research, various studies have disclosed sericin’s therapeutic effects, including its anti-inflammatory [162,163] and antioxidative [164] activities. Moreover, it has been reported that sericin exhibits a great impact in anticancer research due to its cytotoxic activity, and also, it has been used for nanocarrier formulations in anticancer therapy [165]. In Table 4 are listed the studies that used sericin as a nanocarrier system to deliver target antitumoral agents.

Niu et al. (2021) [178] investigated the antitumoral activity of distinct concentrations of sericin on two triple-negative human breast cancer cell lines, this being one of the most aggressive type of cancers. After applying the sericin treatment, the cell viability was analyzed by using the MTT assay, and the results showed that sericin exhibits an antitumoral effect in a dose-dependent way. Additionally, the colony formation rate and proliferation were suppressed by sericin. Furthermore, flow cytometry was used to investigate the apoptosis and cell cycle progress, and the results revealed that the natural compound led to cell cycle arrest, specifically in the G0/G1 phase. These results were confirmed by assessing the expression level of several target proteins, including cyclin D1, Cdk4, E2F3, P21, and P27. Additionally, the apoptotic rate was evaluated, and it validated the tumoral cell apoptosis caused by sericin. In addition, the induced tumor cell apoptosis was investigated by analyzing the level of expression of several apoptosis-related proteins, specifically Bax, Cyto-C, and Bacl-2. The first two proteins were upregulated, while the last one was downregulated by applying sericin as the treatment, confirming the induced cellular apoptosis. In another study, Kumar et al. (2019) evaluated sericin’s antitumoral activity on three different tumoral cell lines, specifically squamous carcinoma (A431), tongue carcinoma (SAS), and breast adenocarcinoma (MCF-7). By applying 1 mg/mL of silk sericin treatment, the viability of the cancer cells was not affected, but by increasing the concentration by 4 mg/mL, the cell viability was negatively impacted in all tumoral cell lines. Comparing the normal cells’ and tumoral cells’ viability after treating them with silk sericin, the authors revealed that sericin exhibits a greater cytotoxicity to tumoral cell lines than healthy cells [179].

## 5. Mulberry as an Alternative Anticancer Approach

Numerous studies have certified the health-promoting benefits of the mulberry plant—more specifically, the medicinal value of mulberry leaves, root, bark, or fruits [63]. One of the greatest bioactivities of the mulberry is the antitumoral effect.

Shu et al. (2020) [180] isolated and purified guangsangon E, a phenolic compound that is found in *M. alba*, in order to evaluate its antitumoral activity against respiratory cancer. Two cancer cell lines were used, specifically a lung cancer (A549) and a nasopharyngeal carcinoma cell line (CNE1), and were treated with distinct concentrations of guangsangon E for 24, 48, and 72 h. In both tumoral cell lines, guangsangon E displayed antitumoral activity and significantly negatively impacted the colony formation. Analyzing the type of cell death induced by the phenolic compound, the authors revealed that, in the treated cells, the ratio of apoptotic cells was 35.4%. Moreover, these results were confirmed after the examination of the expression level of two key players for tumor progression, namely *Bax* and *Bcl-2*. Its expression levels were significantly decreased in the cells that received guangsangon E treatment. Furthermore, the impact of the phenolic compound on a specific autophagosome marker (LC3) was assessed. Their data showed that guangsangon E and the ration levels of LC3 were remarkably increased, revealing the influence of this element on enhancing the autophagy flux in tumoral cell lines. The autophagy and apoptosis connection was investigated by analyzing through immunoprecipitation the interaction of the Bcl-2 protein and an antiapoptotic protein named Beclin 1. The interaction of the two elements was diminished by applying the guangsangon E treatment; therefore, the autophagy process was stimulated by dissociating the antiapoptotic protein from Bcl-2. Moreover, the authors exhaustively analyzed the role of autophagy in the antitumoral effect of guangsangon E by using an autophagy inhibitor, chloroquine. Its association with guangsangon E enhanced the tumoral cells’ viability, suggesting the fact that the cell death was caused by autophagy. The next step was the knocking down of an autophagy-related gene, *Atg5*, and evaluating the viability of genetically modified cells after the treatment was applied. The knockdown of the target gene led to a lower apoptosis rate in the presence of guangsangon E. These results indicate that, through autophagy, this phenolic compound exhibits great antitumoral activity.

One of the most important secondary metabolites produced by mulberry leaves is chalocomoracin. It represents a defense system against fungal germination. It is well documented that it exhibits several bioactivities, including antibacterial and antiviral effects. Han et al. (2018) [181] explored the anticancer activity of chalocomoracin both in vitro and in vivo; in addition, its molecular mechanism was studied. The cytotoxic effect was studied on two human prostate cancer cell lines, LNCaP and PC-3, and one breast cancer cell line, MDA-MB-231. Their data showed that, by treating the cell lines with the target compound, the cell viability was significantly decreased. Interestingly, in two cancer cell lines, PC-3 and MDA-MB-231, the cell death was not triggered by apoptosis but by extensive cytoplasmic vacuolation, this event being distinctive for the paraptosis pathway. Additionally, their findings indicate that chalocomoracin enhanced the endoplasmic reticulum stress but also promoted mitophagy. In addition, the expression level of a key regulator, PINK1, was explored. When mitochondrial damage occurs, high ratios of PINK1 are registered; further, its role in paraptosis was highlighted when the chalocomoracin was applied on the cell lines and PINK1 was upregulated. Additionally, by using nude mice, the impact of chalocomoracin on breast cancer was evaluated in vivo. By administering different doses of the target compound, a significant tumor inhibition was observed up to 54%. The molecular analyses revealed that the treatment also upregulated PINK1 in vivo.

In 2021, Dalkiliç et al. [182] explored in a novel study the cytotoxic potential of M. nigra fruit extract by analyzing its effects on two human cancer lines, specifically MDA-MB-231 (breast cancer) and PC3 (prostate cancer). The authors applied the mulberry extract on the cell lines for three days by using five different concentrations: 1%, 1.33%, 2%, 4%, and 10%, respectively. For measuring the cell viability, the MTT assay was used, and the results revealed that the mulberry exhibits a cytotoxic effect in a concentration-dependent manner. When it comes to the prostate cancer cell line, the cell viability was affected only by applying the mulberry extract of 10% concentration. On the other hand, when applying mulberry extract of lower concentrations, no cytotoxic effect was registered. As for the breast cancer cell line, similar results were registered—the highest concentration led to the most powerful cytotoxic effect; however, lower anticancer activity was reported at 1%, 1.33%, 2%, and 4%, respectively. Moreover, when comparing the anticancer effect of the same concentrations of doxorubicin and mulberry extract, the cytotoxic effect of the fruit extract was higher than the outcome of using the chemotherapy drug.

In another study, the flavonoids were extracted from mulberry leaves (*M. alba*) to explore its potential as an anticancer treatment. Moreover, in this study, the authors explored the interaction between the flavonoids and DNA. Calf thymus DNA was incubated with various concentrations of the target compounds and analyzed after 45 min by using a spectroscopic analysis. The results showed that the extracted flavonoids inhibited tumor growth through DNA modifications by binding to the sugar–phosphate backbone. However, the in vivo experiments did not confirm the flavonoids’ effect in inhibiting tumor growth but increased the lifespan of cancerous mice [183]. In Table 5 are listed the novel studies that confirmed the antitumoral effects of different compounds that are found in the mulberry.

## 6. Conclusions

Cancer remains a major public health burden, and its incidence is continually growing, despite the massive effort that is being made by the scientific community. Even if, in the pharmaceutical industry, a wide range of therapeutic agents is available, they face numerous impediments. In this regard, researchers worldwide are constantly searching for natural compounds that could be a source for novel leads in anticancer research. Sericulture’s applicability is no longer limited to the textile industry; it displays a tremendous impact on various science-related fields, including cancer research. The main reason that brought *B. mori* into the spotlight is the great applicability of SF in the biomaterials area. Numerous studies have used this polymer to overcome certain drawbacks that most of the cancer therapeutic agents exhibit—for instance, drug’s high cytotoxicity on normal cells or its low availability. In this direction, a wide range of researchers have used SF as a drug carrier and demonstrated its feasibility to successfully deliver target agents that exhibited cytotoxicity against various cancer cell lines and achieved long-term release. SF’s applicability in cancer research and therapy is not only due to its role as an anticancer drug delivery strategy, but it is a commonly used biomaterial for the development of 3D tumor models. The 3D models exhibit superior biomimetic properties compared to the 2D models, leading to a better understanding of cancer molecular mechanisms, and play a crucial role in the development of novel anticancer strategies.

Considering the great potential of plants as a rich reservoir of various bioactive elements, tremendous efforts are being made to develop anticancer plant-based therapeutics. Moreover, a wide range of anticancer agents that are frequently used in clinical practice originate from plants, such as irinotecan and paclitaxel. It is well documented that the mulberry composition includes various bioactive elements that make mulberry a great functional food. In this direction, due to its complex composition, numerous studies have certified the anticancer effects of the mulberry and underlined its potential as an anticancer alternative therapy.

## Figures and Tables

**Table 1:** Clinically significant silk-based biomaterials.

Biomaterial	Target	Clinical Significance	Experimental Model	Reference
Silk fibroin (SF) hydrogel	Quiescent ventricular cardiomyocytes	Biological pacemakers cells production intended for human use	Rats	[77]
Electrically conductive SF-based scaffolds	Peripheral nerve tissue	Peripheral nerve injury regeneration	In vitro	[78]
SF nanofibers	Kidney tissue	Renal protection	Mice	[79]
Fluorescent SF bioink-hydrogel printing	Tissues	To keep track of encapsulated cell and the evaluation of hydrogel degradation	Mice	[80]
Biomimetic SF-based biomaterials	Whole blood and platelet	Blood contacting devices production for diagnostic and therapy	In vitro; ex vivo	[81]
SF hydrogel	Human Wilms tumor	Drug delivery	In vitro	[82]
SF/hyaluronic acid injectable hydrogels	Articular cartilage	Cartilage defects regeneration	Mice	[83]
SF microneedles	Prostate gland	Drugs sustained release	Rats	[84]
SF discs	Mucosal tissue	Viral prevention and drug delivery	In vivo, ex vivo	[85]
SF xerogels	Hormones	Drug delivery	In vitro	[86]
Chitin/SF nanoribbons	Neural tissue	Neural tissue regeneration	In vitro	[87]
SF nanofibers	Cardiac tissue	Cardiac tissue regeneration	In vitro	[88]
SF scaffolds	Vascular tissue	Long-distance vascular defect restauration	Rabbits	[89]
SF sponges	Soft tissues	Wound injuries regeneration	In vitro	[90]
Photo-lyogels SF-based	Tissues	Tissue engineering	In vitro	[91]
Microneedles patches SF-based	Blood	Treatment of Insomnia	Rat	[92]
SF dressings	Cutaneous wounds	Burn wound healing	Rat	[93]
Cryo-sponges SF-based	Bone marrow mesenchymal stem cells	Exosome therapy	Mice	[94]
Vascular graft SF based	Abdominal venous system	Replacement of abdominal venous system	Rat	[95]
SF sponges enriched with peptide	Cartilage and bone tissue	Biological functionality improvement of materials that are used for prosthetic devices	Ovine	[96]
SF/Polyurethane patch grafts	Vessels	Vascular diseases treatment	Rat	[97]

**Table 2:** Bioactivities of mulberry leaf extracts and their clinical significance.

Formulation	Bioactivity	Clinical Significance	Experimental Model	Reference
Powder	Antioxidant and anti-inflammatory	Alleviation of hepatic injuries	Fish	[117]
Flavonoids extract	Anti-obesity	Obesity prevention and therapy	In vitro	[118]
Moracin N extract	Antioxidant	Maintenance of oxidation-antioxidation balance	In vitro	[119]
Protein hydrolysates	Anti-inflammatory	Prevention and treatment of colitis	Mice	[120]
Mulberry leaves dietary supplementation	Anti-obesity	Regulation of lipid metabolism	Pig	[121]
Mulberry leaves extract	Hypoglycemic, antioxidative, cardioprotective	Protection against diabetic myocardium	Mice	[122]
Moracin N	Antioxidative, neuroprotective	Protection against neurotoxicity	In vitro	[123]
Mulberry leaf polysaccharides	Anti-obesity	Obesity prevention and treatment	In vitro	[124]

**Table 3:** Fibroin-based carriers for various cancer types.

Carrier Formulation	Loading	Cancer Type	Study Type	Preparation Method	Reference
Nanoparticles	Anastrozole	Breast cancer	In vitro	Solvent change	[134]
Nanoparticles	Doxorubicin	Not specified	In vitro, in vivo	Gas diffusion method	[135]
Silk fibroin (SF)/Chitosan nano- and microparticles	Carboplatin	Breast cancer	In vivo	Ionotropic gelation	[136]
Nanoparticles	Cisplatin	Lung cancer	In vivo	Electrospray	[137]
SF/Gelatin sponges	Curcumin, docosahexaenoic acid	Cervical cancer	In vitro	Freeze-drying; glutaraldehyde cross-linking	[138]
Polyethylenimine-modified SF nanoparticles	Doxorubicin, surviving siRNA	Breast cancer	In vivo	Dropwise addition of acetone in SF solution (2% w/v)	[139]
Nanoparticles	Rosmarinic acid	Cervical carcinoma and breast cancer	In vitro	Dissolution of SF in ionic liquid by using high power ultrasound	[140]
Magnetic nanoparticles SF	Doxorubicin	Breast cancer	In vitro	Salting-out precipitation of potassium phosphate and including hydrophilic magnetic iron oxide nanoparticles into the phosphate solution	[141]
Hydrogels	Iodine	Osteosarcoma	In vivo	Mixing Sf solution, polyethylene glycol 400, polyvinylpyrrolidone iodine, and meglumine diatrizoate	[142]
Nanoparticles	Paclitaxel	Colon cancer, breast cancer	In vitro	Desolvation method	[143]
SF wafers	Etoposide	Neuroblastoma	In vivo	Utilization of a bench-top compression press	[144]
SF foam	Dinutuximab	Neuroblastoma	In vitro	Mixing SF, glycerol and dinutuximab	[145]
Nanoparticles	Triptolide, celastrol	Pancreatic cancer	In vitro	Desolvation method	[146]
Nanoparticles	5-fluorouracil	Adenocarcinoma	In vitro	Nanoprecipitation	[147]
Gene delivery system	Inhibitor of growth 4 and interleukin-24 co-expression plasmid	Lung cancer	In vitro	Freeze-drying	[148]
Nanoparticles	Docetaxel	Breast cancer	In vitro	Nanoprecipitation	[149]
SF meshes	Camptothecin	Colon cancer	In vitro	Electrospinning process	[150]
Nanoparticles	Tamoxifen	Breast cancer	In vitro	Desolvation method	[151]
SF/Selenium nanoparticles	Fingolimod	Thyroid cancer	In vivo	Freeze-drying	[152]

**Table 4:** Silk sericin carriers for anticancer therapy.

Carrier Formulation	Loading	Cancer Type	Study Type	Reference
Nanoparticles	Doxorubicin	Breast cancer	In vitro	[166]
Sericin/poly(γ-benzyl-l-glutamate nanomicelles	Doxorubicin	Hepatocellular carcinoma; breast cancer	In vitro; in vivo	[167]
Nanoparticles	Chlorin e6	Breast cancer	In vitro; in vivo	[168]
Microparticles	Metal-organic networks; Doxorubicin	Lung cancer	In vitro; in vivo	[169]
Sericin/Dextran hydrogel	Doxorubicin	Melanoma	In vitro; in vivo	[170]
Sericin/Synthetic poly(γ-benzyl-l-glutamate	Paclitaxel	Gastric cancer	In vitro; in vivo	[171]
Sericin-Montmorillonite nanoparticles	Doxorubicin	Hepatocarcinoma	In vitro	[172]
Zein/Sericin nanoblends	5-Fluorouracil	Breast cancer; colon carcinoma	In vitro	[173]
Nanoparticles	Curcumin	Not specified	In vitro; in vivo	[174]
Nanoparticles	Resveratrol	Colorectal carcinoma	In vitro	[175]
Albumin-Sericin nanoparticles	Small interfering RNA	Laryngeal cancer	In vitro	[176]
Nanoparticles	Doxorubicin	Breast cancer	In vitro; in vivo	[177]

**Table 5:** The antitumoral activity of mulberry compounds.

Mulberry Species	Compound	Target Cancer	Molecular Cell Death Basis	Reference
*M. nigra*	Total flavonoids, phenolic compounds	Colon cancer	Enhanced expression level of *Bax*/*Bcl-2* genes and a decrease expression ratio of *p53* gene	[184]
*M. alba*	Anthocyanins	Thyroid cancer	Apoptosis and autophagy	[185]
*M. alba*	Albanol B	Lung cancer	Mitochondrial reactive oxygen species production	[186]
*M. alba*	Cyanidin-3-glucoside	Breast cancer	Caspase-3 cleavage, DNA fragmentation	[187]
*M. alba*	Polyphenols	Hepatocellular carcinoma	Apoptosis; autophagy, PI2K/Akt pathway	[188]
*M. alba*	Indole acetic acid	Cervical cancer	Caspase-8 and -9 activation	[189]
*M. nigra*	Phenolic compounds	Prostate adenocarcinoma	Enhanced caspase activity; low mitochondrial membrane potential	[190]
*M. alba*	Lectin	Breast cancer	Caspase dependent pathway	[191]
*M. alba*	Polysaccharides	Breast cancer	Not applicable	[192]
*M. nigra*	Morniga G, chalcone 4 hydrate	Colorectal cancer	Not applicable	[193]

## Data Availability

Not applicable.
